# A prospective observational study of machine translation software to overcome the challenge of including ethnic diversity in healthcare research

**DOI:** 10.1002/nop2.13

**Published:** 2015-01-29

**Authors:** Rachel M. Taylor, Nicola Crichton, Beki Moult, Faith Gibson

**Affiliations:** ^1^ School of Health & Social CareLondon South Bank UniversityLondonUK; ^2^ Cancer Clinical Trials UnitUniversity College London Hospitals NHS Foundation TrustLondonUK; ^3^ Great Ormond Street Hospital for Children NHS Foundation TrustLondonUK

**Keywords:** Communication, consent, outcomes, translation

## Abstract

**Aim:**

This study investigates whether machine translation could help with the challenge of enabling the inclusion of ethnic diversity in healthcare research.

**Design:**

A two phase, prospective observational study.

**Methods:**

Two machine translators, Google Translate and Babylon 9, were tested. Translation of the Strengths and Difficulties Questionnaire (SDQ) from 24 languages into English and translation of an English information sheet into Spanish and Chinese were quality scored. Quality was assessed using the Translation Assessment Quality Tool.

**Results:**

Only six of the 48 translations of the SDQ were rated as acceptable, all from Google Translate. The mean number of acceptably translated sentences was higher (*P *= 0·001) for Google Translate 17·1 (sd 7·2) than for Babylon 9 11 (sd 7·9). Translation by Google Translate was better for Spanish and Chinese, although no score was in the acceptable range. Machine translation is not currently sufficiently accurate without editing to provide translation of materials for use in healthcare research.

## Introduction

World migration is higher than ever, not just people voluntarily looking for a better life but also as a result of conflict and environmental disasters (International Organization for Migration 2013). This increase can be seen in the change in Census statistics for England and Wales where 87·5% of the population were white British in 2001 (Office for National Statistics 2004), which decreased to 80·5% in 2011 (Office for National Statistics 2012). This poses a challenge for public services, because providing information that is accessible to all society therefore requires written documents to be translated in to a wide range of languages and this is costly. In the UK in 2011, the National Health Service (NHS) spent £23·3 million pounds on translation, which has led some to question whether documents should be available solely in simple English rather than providing translations into multiple languages (Gan 2012).

However, to ensure health care is equitable information needs to be available in languages other than just the country's native language. Similarly, for research to be generalizable, it needs to include participants representing the whole population; information solely in the native language would not facilitate this. Verbal information can be conveyed through interpreters, but the process of ‘informed consent’ depends on written information being available, to be used in conjunction with verbal explanations, to facilitate reflection before deciding whether or not to take part (Dixon‐Woods *et al*. 2007, Wynia & Osborn 2010). In the UK, the NHS Research Ethics Committees require justification if only English literate participants are to be included in a study (http://www.hra.nhs.uk/documents/2013/08/language-and-exclusion.pdf). It is, however, a challenge to balance fairness and the desire to have a sample representing the population with the limitations of resources to enable comprehensive translation. One solution to overcome the cost of translating research documents by human translators could be through the use of machine/computerised translation software.

This study focuses on two aspects of research where machine translation may be useful: patient information sheets and data collection through survey methods. However, the results will have translational application to clinical practice where conveying information and gathering experience data is part of standard care.

## Background

Since the 1940s, there has been interest in developing automated translation (Kirchhoff *et al*. 2011). However, it was not until the 1990s that the technology became sophisticated enough for effective translation software to be developed. Machine translators work through referencing the source text to a corpus, i.e. a ‘body of text in the source language paired with its translation in the target language’ (Kirchhoff *et al*. 2011). The challenge for machine translation developers was identifying large enough corpora for comparison.

Availability of machine translation does not necessarily equate to higher quality translation. To provide quality translation, various factors need to be taken into consideration e.g. co‐reference structure, semantics of the source language, text style, idiomatic expressions and syntax of the source language and transcription language (Och 2005). Garcia (2010) suggested that rather than assessing quality of translation using criteria for assessing human translation, a more pragmatic approach should be taken so that the translation is understood ‘by the educated bilingual rather than by the professional translator’ (pp. 10). This pragmatic approach may be sufficient for translating non‐essential information but is unsuitable for conveying information in health care, especially when informed consent is required (Hablamos Juntos 2009b). Assessment of quality therefore needs to reflect the context of the source document but also the simplicity of the language.

There are two sources of machine translators: commercial software and free machine translators available through the Internet. The quality of the translation provided through both sources is variable. Commercial translation software is evaluated annually by an independent organization (Top ten reviews 2013). The top commercial translation software for 2012 is presented in Table 1. In competition with commercial translation software, several free online translation portals have become available, e.g. Google Translate (http://translate.google.co.uk). Similar to commercial software, these have also been evaluated to give a ranking of quality (Table 1) (Hampshire & Salvia 2010).

**Table 1:** Global Internet Ranking Results for machine translators

Rank	Commercial software^a^	Free machine translators^b^
1	Babylon 9^c^	Google translate
2	Power translator	Babylon^c^
3	Prompt	Reverso
4	WhiteSmoke	Bing
5	Translate personal	Babelfish^d^
6	Prompt personal	Systrans
7	Translution	Prompt
8	LingvoSoft translator	Worldlingo
9	IdiomaX	Intertran
10	Ace translator	Webtrance

a Top ten reviews (2013).

b Hampshire and Salvia (2010).

c Babylon has a commercial version but also has a free version that does not contain all the features available in the commercial software.

d Yahoo version.

Interestingly, while there is a need for quick, cheap and accurate translation of documents into multiple languages, there is limited evaluation of translation software used in health care. Google Translate has been assessed for ‘information retrieval effectiveness’ and was found to be 88% effective for making bilingual Internet searches of non‐medical information (Savoy & Dolamic 2009). Rather than relying on machine translators to provide perfect translation, the software has been evaluated to assess the quality of translation‐with‐proof reading compared to human translation direct from the source text. Translation‐with‐proof reading is the process where rather than translating text from the source document, the translator reviews translated text and edits errors of grammar or punctuation, spelling mistakes or non‐idiomatic expressions. This was found to produce equivalent quality but again, this was not in healthcare literature (Garcia 2010). The only research conducted in health care has been a feasibility study of machine translation for translating public health material, comparing human post‐editing to human‐only translation (Kirchhoff *et al*. 2011). Similar to Garcia, there was equivalent quality in the translation, with the added benefit of post‐editing being quicker (between 15–53 minutes per document compared to 2–7 days for human‐only translation). This study will investigate whether machine translation could help with the challenge of including ethnic diversity in healthcare research and the implications for clinical practice.

## Methods

### Design

This was a two phase, prospective observational study. Phase 1 was to evaluate the quality of back translation into English of validated translations of a validated questionnaire using machine translators. Phase 2 used lay human translators to evaluate the quality of the translation of a participant information sheet from English into other languages using machine translators.

The terms forward and backward translation describe the recognized process of ensuring the accuracy of translated documents (Acquadro 2004). Documents are translated from source language to target language (forward translation) and then translated documents are translated back into the language of the original text (backward translation).

To account for variation in quality of different machine translators, two were selected for the evaluation: one commercial and one freely available on the Internet. Google Translate (free) and Babylon 9 (commercial) were chosen as they both ranked highest (Table 1).

### Measure of translation quality

Several scoring scales have been developed to rate quality of translation, e.g. clarity and fidelity (Hampshire & Salvia 2010), adequacy and fluency (Kirchhoff *et al*. 2011). However, these have had limited psychometric testing and the vague classification (e.g. ‘complete gibberish’) are open to interpretation. The Translation Quality Assessment (TQA) Tool was developed to go beyond ‘good’ or ‘bad’ translation and to provide detailed analysis of translation deficiencies (Hablamos Juntos 2009a). The translated text is assessed by professionals or academics with training in translation in four key categories: target language, textual and functional adequacy, non‐specialized content and specialized content. Each category is scored on four levels ranging from, for example, 0 = ‘reads similar to target text’ to 3 = ‘text is extremely difficult to read’. The sum of the four categories gives a total score (range 0–12), the higher the score reflecting the poorer the quality of the translation. The translated text is reviewed four times, each time evaluating one of the categories. For the two categories evaluating the meaning, the review is conducted in comparison to the English version of the source document so the reviewer can evaluate how well the translation communicates the context of the translation. The TQA Tool has good content validity (Hablamos Juntos 2009a) and inter‐rater reliability reported for Spanish (r = 0·934) and Chinese (r = 0·78) (Colina 2008, 2009).

### Phase 1

To evaluate backward translation, it was necessary to source a document developed in English that had multiple validated translations readily available. The Strengths & Difficulty Questionnaire (SDQ) is a behavioural screening tool for children (Goodman *et al*. 1998), which is freely available through the Internet (http://www.sdqinfo.org/). It contains 25 statements, such as ‘I try to be nice to other people. I care for their feelings’. The English version is validated for children aged 11–17 years but has a Flesch reading ease of 91·7% (90–100% is interpreted as being understood to an average 11 year old) and Kincaid–Flesch grade of 2 (equivalent of USA school grades so understandable to those aged 7–8 years), indicating it is written in very simple English (Franck & Winter 2004). The SDQ has validated translations in over 50 languages. Twenty‐four translations in languages that were available in both Babylon 9 and Google Translate were selected to be translated back into English by each machine translator. The quality of translation was evaluated independently by two researchers (RT, NC) using the TQA Tool.

### Phase 2

Forward translation was evaluated through translating a document from English into another language. A dummy participant information leaflet (source text; Appendix 1) was written in line with guidance from the UK National Research Ethics Service (National Patient Safety Agency 2011). The information leaflet was 600 words in length and contained only the core components that are recommended for inclusion in all participant information leaflets. The language and grammar was edited until it had a Flesch reading ease of >70% and Kincaid–Flesch grade of ≤7 (Franck & Winter 2004).

#### Participants

A convenience sample of participants was recruited though a cross‐faculty email at a UK University and through a snowball sampling technique. Participants were eligible to take part if they were: fluent in Spanish or Chinese and fluent in written/spoken English. These languages were chosen to reflect the validation of the TQA Tool but also they represented an easy (Spanish) and complex (Chinese) language for those whose first language is English (Foreign Service Institute 2013). The aim was to recruit 6–10 participants per language. After completing the translation review, participants were given a £20 gift token to compensate for their time. The study was approved by the University Research Ethics Committee.

#### Procedure

The source text was translated by each machine translator into Spanish and Chinese (simplified). It was stressed to participants that they were not being tested, rather it was the quality of the translation being assessed and therefore they should do this without conversing with colleagues. Participants were given half the source text translated in Google Translate and half by Babylon 9 (each half of the text had been set up so that the length of text and Flesch reading ease were similar). Each participant was asked to assess each half of the source document separately using the TQA Tool and to make any comments they felt would add to the review.

### Phase 1 and 2 analysis

The descriptions for each category of the TQA Tool were coded 0 (best description)–3 (worst description) to give category scores and an overall score computed for the sum of the category scores: 0 (perfect translation quality)–12 (poorest level of quality). Translations were deemed acceptable if the total score was 0–3. Data were analysed using descriptive statistics. The Wilcoxon signed rank test was used to compare TQA total scores for Google Translate and Babylon 9. Paired *t*‐tests were used to compare the mean number of acceptable sentences. Qualitative content analysis was used for analysing comments, which were used to clarify the scores from the TQA Tool.

## Results

### Phase 1

The TQA scores are shown in Table 2. Google Translate performed better than (lower score) Babylon 9 in every category in most languages, although there were examples where Babylon 9 scored better. For example, in ‘target language’ Babylon 9 rated better than Google Translate for Japanese back‐translation. However, Babylon 9 did not score ‘0’ (best possible) for any category in any language, which Google Translate did for between 2–6 languages per category.

**Table 2:** Quality of the backward translation of the Strengths and Difficulties Questionnaire (SDQ) into English

Language	Target language^a^		Textual & functional adequacy^a^		Non‐specialized content^a^		Specialized language^a^		TQA total score^b^	
	Babylon	Google	Babylon	Google	Babylon	Google	Babylon	Google	Babylon	Google
Arabic	3	2	3	2	3	2	3	1	12	7
Bulgarian	3	2	2	2	2	1	3	1	10	6
Chinese (simple)	3	2	2	2	2	2	3	2	10	8
Czech	1	1	1	2	1	2	2	2	5	7
Danish	3	0	2	0	3	1	3	1	11	2
Dutch	1	0	1	1	1	1	1	1	4	3
French	2	0	2	0	2	1	2	1	8	2
German	3	0	2	0	1	1	2	1	8	2
Hebrew	3	1	2	2	1	2	2	1	8	6
Hungarian	3	2	3	2	3	2	3	2	12	8
Italian	2	2	1	2	1	2	2	2	6	8
Japanese	1	3	2	2	2	2	2	2	7	9
Korean	3	3	3	2	3	2	3	2	12	9
Norwegian (Bokmal)	1	0	2	0	1	0	2	0	6	0
Polish	2	1	2	2	2	1	2	2	8	6
Portuguese	2	2	1	1	1	2	2	3	6	8
Romanian	2	1	1	2	1	2	2	2	6	7
Russian	3	2	2	2	3	2	2	2	10	8
Serbian	3	3	2	2	2	2	3	2	10	9
Spanish	1	1	1	1	1	1	1	1	4	4
Swedish	2	0	1	1	2	0	2	0	7	1
Thai	3	1	2	2	2	1	2	2	9	6
Turkish	3	2	3	2	3	3	3	3	12	10
Ukrainian	3	2	3	2	3	3	3	3	12	10

a Scored 0–3.

b Scored 0–12 (the higher the score, the worst the translation quality).

The median total TQA score from the back‐translation through Babylon 9 was 8 (range 4–12), with none of the back translations evaluated as acceptable (score 0–3). In 10 languages (Arabic, Danish, Dutch, Hungarian, Japanese, Polish, Serbian, Swedish, Turkish, Ukrainian) there were words that were translated into nonsense or remained in the source language. The median total TQA score translating back through Google Translate was 7 (range 0–10), with Norwegian (Bokmal) evaluated as having perfect translation and Danish, Dutch, French, German and Swedish rated as having acceptable translations. In six languages (Hungarian, Italian, Japanese, Korean, Turkish, Ukrainian), there were words that were translated into nonsense or remained in the source language. Comparison of the TQA total score for Babylon 9 and Google Translate using Wilcoxon signed rank test indicated Google had significantly lower TQA total scores, *P *=* *0·002.

**Figure 1:**
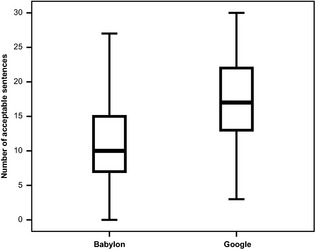
Boxplot comparing the distribution of the number of acceptable sentences from backward translation of the 33 statements of the SDQ in both Babylon and Google for 24 different languages

The SDQ contains 33 sentences; the number of sentences that were rated as an accurate and appropriate rendition of terminology is shown in Table 3. The mean number of accurate sentences in the Babylon 9 backward translations was 11 (sd 7·9; range 0–27) and in the Google Translate backward translations were 17·1 (sd 7·2; range 3–30). Using a paired t‐test, there were significantly more acceptable sentences by Google Translate (*t *=* *3·945, d.f. = 23, *P *=* *0·001). Figure 1 compares the distribution of the acceptable sentences resulting from the Google Translate translation of the SDQ with the distribution resulting from the Babylon 9 translation. The higher numbers of acceptable sentences arising from Google Translate is apparent; the median from Babylon 9 is clearly below the lower quartile of the Google boxplot.

**Table 3:** Number of sentences in the Strengths and Difficulties Questionnaire (SDQ) rated as acceptable (*N* = 33)

Language	Number (%) acceptable sentences	
	Babylon	Google
Arabic	8 (24)	6 (18)
Bulgarian	10 (30)	12 (36)
Chinese (simple)	10 (30)	5 (15)
Czech	1 (3)	17 (52)
Danish	27 (82)	28 (85)
Dutch	7 (21)	23 (70)
French	27 (82)	20 (61)
German	12 (36)	24 (73)
Hebrew	14 (42)	21 (64)
Hungarian	1 (3)	16 (48)
Italian	16 (48)	16 (48)
Japanese	11 (33)	3 (9)
Korean	0	9 (27)
Norwegian (Bokmal)	14 (42)	30 (91)
Polish	9 (27)	18 (55)
Portuguese	20 (61)	15 (45)
Romanian	13 (39)	20 (61)
Russian	10 (30)	18 (55)
Serbian	7 (21)	13 (39)
Spanish	22 (67)	26 (79)
Swedish	17 (52)	27 (82)
Thai	8 (24)	17 (52)
Turkish	0	14 (42)
Ukrainian	0	13 (39)

**Figure 2:**
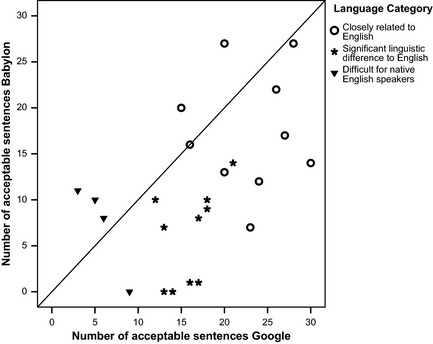
Scatterplot showing, for the backward translation of the SDQ, the number of acceptable sentences from Google translate against number of acceptable sentences from Babylon for 24 different languages. The line of equality (y=x) is shown on the figure

Figure 2 shows for each of the 24 languages the number of acceptable sentences from Google Translate translation plotted against the number of acceptable sentences from Babylon 9. For each language, the complexity of that language for native English speakers is indicated. Class 1 languages are closely related to English and tend to score more acceptable sentences in both translators, Class 2 are languages showing significant linguistic difference to English while Class 3 languages are considered difficult for native English speakers (Foreign Service Institute 2013). The better performance of Google Translate compared to Babylon 9 for Class 1 and Class 2 languages is evident in Figure 2 from most points being below the line of equality (*y* = *x*); for Class 3 Babylon 9 appear to be superior, though neither translator is performing well for these languages.

### Phase 2

A total of 16 volunteers participated in the evaluation of the forward translation into Spanish (*n *=* *6) and Chinese (*n *=* *10). All were native Spanish and Chinese educated to at least first degree. No other details of participants were obtained to assure anonymity. Total TQA and qualitative comments are presented together; comments are [uncorrected] verbatim quotes.

The component and total TQA scores are shown in Table 4. While there was considerable agreement among reviewers of the Spanish translation, there was wide variation in the Chinese assessments. Babylon 9 and Google Translate were rated equally for the Spanish translations based on ‘textual and functional adequacy’ and ‘specialized content’. Google Translate was rated better for the other components and also had a lower total score. Similarly, Google Translate was rated better in all the components and total score of the assessment of the Chinese translations. While Google Translate outperformed Babylon 9 for both languages, median scores were >3 so were not rated as being acceptable. Comparing the TQA total score for Google Translate with Babylon 9 for Spanish using Wilcoxon signed rank test, there was no difference between the machine translators (*P *=* *0·102). However, for the Chinese translation there was a significant difference (*P *=* *0·007) with Google Translate being significantly better than Babylon 9.

**Table 4:** Evaluation of the translations of the source text

Language	Target language^a^		Textual & functional adequacy^a^		Non‐specialized content^a^		Specialized language^a^		TQA total score^b^	
	Babylon	Google	Babylon	Google	Babylon	Google	Babylon	Google	Babylon	Google
Spanish										
Median	2	1	1	1	2	1·5	1	1	5·5	4
Range	1–3	0–1	1–2	0–2	1–3	0–2	All 1	0–2	5–9	0–6
Chinese (simple)										
Median	2	1	2	1	2·5	1	2	1	8	5
Range	1–3	1–2	0–3	0–2	1–3	1–2	0–3	0–2	2–12	2–6

a Scored 0–3.

b Scored 0–12 (the higher the score, the worst the translation quality).

Comments made about the ‘target language’ in Spanish were more favourable about the Google Translate translation, but neither translation was evaluated as being ‘natural’. Similarly ‘functional adequacy’ was noted to be understandable by both software packages but it did not resemble natural Spanish:

I think that although sometimes the text sounds artificial or not natural in Spanish… Much better in text 2 [Google] than in text 1 [Babylon].

‘Non‐specialized’ and ‘specialized’ content were suggested to be flawed because of grammatical errors in the translations in both software packages. The better translation quality by Google Translate Chinese was also reflected in the comments. Although ‘not natural’ or ‘real’ Chinese, the text could be understood by Google Translate:

The text makes me understand the general meaning though there are some grammar errors.

Babylon 9, on the other hand was evaluated as being difficult to understand:

The translated text is so confusing that readers cannot comprehend the general meanings without the original text.

Comments related to the assessment of ‘functional adequacy’ indicated Google Translate had some grammatical errors but these did not detract from the overall meaning. However, Babylon 9 was noted to need:

The whole document needs to be translated completely again.

With regard to content, both software packages reflected well the specialized content.

However, the complexity of English resulted in distortion of the meaning. Through Google Translate the meaning was still understandable. Nevertheless, through Babylon 9, the non‐specialized content was a potential cause of the error in translation:

About 20% of the Text I cannot understand, so I have to skip them. In other words, I understand the text, but I am not Sure 100%.

## Discussion

Our study aimed to evaluate machine translation software for use in healthcare research. We selected the freely available and commercial software that had been rated as providing the most accurate translation. We have found that although Google Translate is superior to Babylon 9, neither machine translator can give consistent translation quality to be of use without editing. However, this may be of significance from a cost perspective. Kirchhoff *et al*. (2011) showed a reduction in translation time from 2–7 days for human translation to 15–53 minutes for editing only. The cost implication can be seen in an example for the cost of translating a 900 word document into Arabic. Full translation costs $169, whereas editing costs $84 (WorldLingo 2013). We therefore suggest a two‐stage process could be employed for translating documents: (1) Translate using a machine translation; (2) Proof reading and editing by a human translator.

It is interesting to note the languages on which machine translators performed better than others. Google Translate was able to provide perfect/acceptable backward translation for several languages, all European and half of these Scandinavian. The reason for this is unclear but may relate to the greater availability of reference text. To translate from a source to target language accurately, a sufficiently large corpus is required in which to reference. It may be that there are larger corpora in these languages than in others. In theory, the Internet gives an ever increasing reference source in many languages, so as the presence of different languages increases through the Internet, the better the translation quality will become. However, for translating information for use in health care, a large enough medical corpora is necessary. This raises an additional problem: the amount of word sense ambiguity there is in the biomedical domain (Zeng‐Treitler *et al*. 2007). In the Unified Medical Language System (UMLS) Metathesaurus, there are over 21,000 ambiguous terms; so a search for BPD can reveal ‘bronchopulmonary dysplasia’, ‘borderline personality disorder’ or ‘biparietal diameter’ (Xu *et al*. 2006). Until there is sufficient word sense disambiguity, machine translation of healthcare literature could remain challenging.

There were several limitations to this study. First, we limited our evaluation to two machine translators that had been reviewed for quality using different methods, neither of which used healthcare language (Hampshire & Salvia 2010, Top ten reviews 2013). Other software programmes may have greater accuracy on this form of literature. Second, both the backward and forward evaluations were reviewed by educated readers. Evaluation by reviewers who have lower reading ability would be important to ensure the context of text was understood by the range of people at whom it is aimed. It is also important to note the evaluators were educated but they did not have training in linguistics so their interpretations of the categories in the TQA will be varied thus scores may be different when judged by an expert. While this may be a limitation, it may also be a strength of the study because participants may reflect the actual population who uses this software. Finally, we wrote the source document in simple English; similarly, the SDQ was also rated as having simple English as assessed through the Flesch and Kincaid–Flesch scales, which are developed to rate English language only. Machine translators may perform better with more complex language as they do not follow linguistic ‘rules’ such as grammar. Despite these limitations, this is the first evaluation of machine translation software for use in healthcare research, assessing the quality of both backward and forward translation of documents used regularly in research. Furthermore, quality was evaluated using a validated tool rather than using classification methods that were open to interpretation.

Our study has some important implications for both research and clinical practice if inaccurate translation is used. Informed consent requires the passage of information written in a simplified way to ensure patients fully comprehend what they are consenting to (National Patient Safety Agency 2011). Inaccurate translation of information can result in sentences becoming overly complicated or worse, altering the meaning. Patients would therefore not be fully informed and could potentially be agreeing to participate in something that was not going to occur. A suggestion published in the British Medical Journal to use Google Translate in clinical practice (Wade 2011) sparked debate not only about the accuracy of translation (Leach 2011) but also the ethical issues of entering patient identifiable information into a system that stores it in its server (Jepson 2011). Furthermore, the impact of inappropriate translation was highlighted by Khalifa (2011), who used it in a consultation with an Arabic speaking lady; the consequence was she thought (incorrectly) that the abdominal pain she presented with was as a result of pregnancy.

## Conclusion and relevance for clinical practice

It has been suggested that the variation in the quality of the translation methods used was due of the lack of formal guidelines on how measures should be translated (Maneesriwongul & Dixon 2004). However, clear guidelines have been published for high quality translation of outcome measures, including those from the International Society for Pharmacoeconomics and Outcomes Research (ISOPOR) (Wild *et al*. 2005), the International Quality of Life Assessment (IQOLA) project (Bullinger *et al*. 1998) and the MAPI Institute (Acquadro 2004). Common to all these guides is the need for forward‐backward translation. While guidance clearly states how translation should be done, they do not state by whom. Reports of translation are published regularly in peer reviewed journals; however, the quality of the method is peer reviewed not the quality of translation. It is important therefore that reports of translation validation that are published in peer reviewed literature clearly specify the source of translation. Machine translation software is not currently accurate enough to provide translation of documents used in research or health care and would not recommend its use without the involvement of professional proofreading and editing.

## Conflict of interest

None of the authors have a conflict of interest to disclose.

## Author contributions

All authors have agreed on the final version and meet at least one of the following criteria [recommended by the ICMJE (http://www.icmje.org/ethical_1author.html)]:

- substantial contributions to conception and design, acquisition of data, or analysis and interpretation of data;
- drafting the article or revising it critically for important intellectual content.

## Appendix 1 Source text

### Evaluating computerised translation software

You are taking part in a study to evaluate the accuracy of computerised translation software. You are assessing a document containing the main details we would include in a patient information sheet. It has been translated by two computer programmes. We have given you the evaluation questionnaire and we would like you to assess each page of this translation separately. If this is not clear then please talk to the researcher.

### Do I have to take part?

No, it is up to you to decide to join the study. We will describe the study and go through this information sheet. If you agree to take part, we will then ask you to sign a consent form. You are free to withdraw at any time, without giving a reason. This will not affect your job at the university.

### What are the risks of taking part?

We do not think that taking part will cause any worries. If you are unhappy while you are taking part in this study, you can stop taking part and this will not have any effect on your employment. We are asking you to give up some of your time to take part in this study. Reviewing each page of this document using the questionnaire will take about 30–60 minutes. You can stop and start as you feel able. If you have some questions after taking part in the study, the researcher will help you.

### What are the benefits of taking part?

We cannot promise the study will help you but the information we get from this study will help improve the quality and quantity of information we can give patients. This will give people, who do not speak English, a chance to take part in more research. The information you give us will determine whether computerised translation software can be used without proof reading by a native language speaker.

Please continue your evaluation using the second form.

### What happens when the research ends?

We will not ask you to do anything else when you have completed the review. We will write the study for publication and present it at a conference. If you are interested, we can keep you updated.

### What if there is a problem?

The research team will address any complaint about the project. If you are still unhappy and want to formally complain, please contact the chair of the Ethics Committee.

### Will my taking part be kept confidential?

All information will be treated in the strictest confidence. Only the research team will have access to the data collected during this study.

### What will happen if I don't want to carry on with the study?

If you withdraw from the study, we will delete your contact details but will use all the data we collected before you stopped. This will not affect your job at the university.

### Who is organizing and funding the study?

The study has been funded by LSBU. This includes the funding for a voucher to thank you for taking part.

### Who has reviewed the study?

All research at LSBU is looked at by an independent group of people, called an Ethics Committee, to protect your interests. This study has been reviewed and approved by the Ethics Committee.

Please look at your assessment before giving it back to the research team to make sure you have put a response in every section. Your comments are also important so we want you to complete these sections as well.

To show us that you have understood the language this has been translated into, please draw a flower on the back of this document.

Thank you for taking part.

## References

[nop213-bib-0001] Acquadro C. 2004 Linguistic Validation Manual for Patient‐reported Outcomes (PRO) Instruments. MAPI Research Trust, Lyon, France.

[nop213-bib-0002] Bullinger M. , Alonso J. , Apolone G. , Leplege A. , Sullivan M. , Wood‐Dauphinee S. , Gandek B. , Wagner A. , Aaronson N. , Bech P. , Fukuhara S. , Kaasa S. & Ware J.E. & for the IQOLA Project Group (1998) Translating health status questionnaires and evaluating their quality: the IQOLA project approach. Journal of Clinical Epidemiology 51(11), 913–923. 9817108 10.1016/s0895-4356(98)00082-1

[nop213-bib-0003] Colina S. (2008) Translation quality evaluation: empirical evidence from a functionalist approach. The Translator 14(1), 97–134.

[nop213-bib-0004] Colina S. (2009) Further evidence for a fundamentalist approach to translation quality evaluation. Target 21(2), 235–264.

[nop213-bib-0005] Dixon‐Woods M. , Ashcroft R.E. , Jackson C.J. , Tobin M.D. , Kivits J. , Burtin P.R. & Samani N.J. (2007) Beyond ‘misunderstanding’: written information and decisions about taking part in a genetic epidemiology study. Social Science & Medicine 65, 2212–2222. 17904716 10.1016/j.socscimed.2007.08.010

[nop213-bib-0006] Foreign Service Institute (2013) Retrieved from http://web.archive.org/web/20071014005901/http://www.nvtc.gov/lotw/months/November/learningExpectations.html on 15 March 2013.

[nop213-bib-0007] Franck L.S. & Winter I. (2004) Research participant information sheets are difficult to read. Bulletin of Medical Ethics 195, 6–13. 15812989

[nop213-bib-0008] Gan S. (2012) Lost in Translation: How Much is Translation Costing the NHS and How Can We Both Cut Costs and Improve Service Provision? 2020Health Retrieved from: www.2020health.org%2Fdms%2F2020health%2Fdownloads%2Freports%2FLOST-IN-TRANSLATION%2FLOST%2520IN%2520TRANSLATION.pdf on 17 January 2015.

[nop213-bib-0009] Garcia I. (2010) Is machine translation ready yet? Target 22(1), 7–21.

[nop213-bib-0010] Goodman R. , Meltzer H. & Bailey V. (1998) The Strengths and Difficulties Questionnaire: a pilot study on the validity of the self‐report version. European Child & Adolescent Psychiatry 7(3), 125–130. 9826298 10.1007/s007870050057

[nop213-bib-0011] Hablamos Juntos (2009a) Assessing Translation: A Manual for Requesters (Tool 6). UCSF Fresno Center for Medical Education & Research, Fresno, CA.

[nop213-bib-0012] Hablamos Juntos (2009b) Creating a Translation Brief For: Informed Consent Forms (Tool 4). UCSF Fresno Center for Medical Education & Research, Fresno, CA.

[nop213-bib-0013] Hampshire S. & Salvia C.P. (2010) Translation and the Internet: evaluating the quality of free online machine translators. Quaderns Review Traditional 17, 197–209.

[nop213-bib-0014] International Organization for Migration (2013) World Migration Report 2013: Migrant Well‐being and Development. International Organization for Migration, Geneva, Switzerland.

[nop213-bib-0101] Jepson P. (2011) RAPID RESPONSE Re: Try Google Translate to overcome language barriers. British Medical Journal. Published November 21/11/11.

[nop213-bib-0015] Kirchhoff K. , Turner A.M. , Axelrod A. & Saavedra F. (2011) Application of statistical machine translation to public health information: a feasibility study. Journal of the American Medical Information Association 18(4), 473–478. 10.1136/amiajnl-2011-000176 PMC3128406 21498805

[nop213-bib-0102] Khalifa S. (2011) RAPID RESPONSE Re: Try Google Translate to overcome language barriers. British Medical Journal. Published November 21/11/11.

[nop213-bib-0103] Leach M.A. (2011) RAPID RESPONSE Re: Try Google Translate to overcome language barriers. British Medical Journal. Published November 16/11/11.

[nop213-bib-0016] Maneesriwongul W. & Dixon J.K. (2004) Instrument translation process: a methods review. Journal of Advanced Nursing 48(2), 175–186. 15369498 10.1111/j.1365-2648.2004.03185.x

[nop213-bib-0017] National Patient Safety Agency (2011) Information Sheets & Consent Forms: Guidance for Researchers and Reviewers. National Research Ethics Service, London.

[nop213-bib-0018] Och F.J. (2005) Statistic Machine Translation: Foundations and Recent Advances. Tutorial at MT Summit 2005, Phuket, Thailand.

[nop213-bib-0019] Office for National Statistics (2004) Census 2001: National Report for England and Wales. TSO, London.

[nop213-bib-0020] Office for National Statistics (2012) Ethnicity and National Identity in England and Wales 2011. TSO, London.

[nop213-bib-0021] Savoy J. & Dolamic L. (2009) How effective is Google's translation service in search? Communications of the ACM 52(10), 139–143.

[nop213-bib-0022] Top ten reviews (2013) Retrieved from http://translation-software-review.toptenreviews.com/on 15 March 2013.

[nop213-bib-0104] Wade R.G. (2011) NHS TRANSLATION SERVICES Try Google Translate to overcome language barriers. British Medical Journal 343, 7217. 10.1136/bmj.d7217 22089735

[nop213-bib-0023] Wild D. , Grove A. , Martin M. , Eremenco S. , McElroy S. , Verjee‐Lorenz A. & Erikson P. (2005) Principles of good practice for the translation nd cultural adaptation process for patient‐reported outcomes (PRO) measures: report of the ISPOR Task Force for translation and cultural adaptation. Value in Health 8(2), 94–104. 15804318 10.1111/j.1524-4733.2005.04054.x

[nop213-bib-0024] WorldLingo (2013) Retrieved from http://www.worldlingo.com/on 15 March 2013.

[nop213-bib-0025] Wynia M.K. & Osborn C.Y. (2010) Health literacy adn communication quality in health care organizations. Journal of Health Communication 15(S2), 102–115. 20845197 10.1080/10810730.2010.499981 PMC3086818

[nop213-bib-0026] Xu H. , Markatou M. , Dimova R. , Liu H. & Friedeman C. (2006) Machine learning and word sense disambiguation in the biomedical domain: design and evaluation issues. BMC Bioinformatics 7, 344. 16822321 10.1186/1471-2105-7-334 PMC1550263

[nop213-bib-0027] Zeng‐Treitler A. , Goryachev S. , Kim H. , Keselman A. & Rosendale D. (2007) Making texts in electronic health records comprehensible to consumers: a prototype translator AMIA Symposium Proceedings 2007, 846–850 PMC2655860 18693956

